# Community health services in European literature: A systematic review of their features, outcomes, and nursing contribution to care

**DOI:** 10.1111/inr.13033

**Published:** 2024-07-29

**Authors:** Valeria Caponnetto, Angelo Dante, Khadija El Aoufy, Maria Ramona Melis, Giulia Ottonello, Francesca Napolitano, Fabio Ferraiuolo, Francesco Camero, Angela Cuoco, Ilaria Erba, Laura Rasero, Loredana Sasso, Annamaria Bagnasco, Rosaria Alvaro, Duilio Fiorenzo Manara, Gennaro Rocco, Maurizio Zega, Giancarlo Cicolini, Beatrice Mazzoleni, Loreto Lancia

**Affiliations:** ^1^ Department of Life, Health and Environmental Sciences University of L'Aquila L'Aquila Italy; ^2^ Department of Health Sciences University of Florence Florence Italy; ^3^ Staff Training Department Careggi University Hospital Florence Italy; ^4^ Department of Health Sciences University of Genoa Genova Italy; ^5^ Ingram School of Nursing McGill University Montreal Canada; ^6^ Direction of Health Professionals “IRCCS Istituto Giannina Gaslini,” Genova Italy; ^7^ Department of Emergency and Admission Policlinic Hospital “IRCSS San Martino,” Genova Italy; ^8^ Department of Biomedicine and Prevention University of Rome Tor Vergata Rome Italy; ^9^ Orthopedic and Traumatology Clinic Orthopedic Institute “IRCSS Rizzoli,” Bologna Italy; ^10^ Bachelor of Science in Nursing, Saint Camillus International University of Health and Medical Sciences Rome Italy; ^11^ Scientific Committee CERSI‐FNOPI, Federazione Nazionale Ordini Professioni Infermieristiche Rome Italy; ^12^ Center for Nursing Research and Innovation Vita‐Salute San Raffaele University Milan Italy; ^13^ Center of Excellence for Nursing Scholarship Rome Italy; ^14^ Faculty of Medicine, University "Our Lady of the Good Counsel" Tirana Albania; ^15^ FNOPI, Federazione Nazionale Ordini Professioni Infermieristiche Rome Italy; ^16^ Section of Nursing and Midwifery, Department of Innovative Technologies in Medicine & Dentistry University "G. d'Annunzio" Chieti ‐ Pescara Chieti Italy; ^17^ Department of Biomedical Sciences Humanitas University Milan Italy

**Keywords:** Community care services, home care services, nursing determinants, nursing homes, patient outcomes, primary care, remote health, systematic review, transitional care

## Abstract

**Background:**

To meet the population's needs, community care should be customized and continuous, adequately equipped, and monitored.

**Introduction:**

Considering their fragmented and heterogeneous nature, a summary of community healthcare services described in European literature is needed. The aim of this study was to summarize their organizational models, outcomes, nursing contribution to care, and nursing‐related determinants of outcomes.

**Methods:**

A systematic review was performed by searching PubMed, CINAHL, Scopus, and Embase in October 2022 and October 2023 (for updated results). Quantitative studies investigating the effects of community care, including nursing contribution, on patient outcomes were included and summarized. Reporting followed the PRISMA checklist. The review protocol was registered on PROSPERO (CRD42022383856).

**Results:**

Twenty‐three studies describing six types of community care services were included, which are heterogeneous in terms of target population, country, interventions, organizational characteristics, and investigated outcomes. Heterogeneous services’ effects were observed for access to emergency services, satisfaction, and compliance with treatment. Services revealed a potential to reduce rehospitalizations of people with long‐term conditions, frail or older persons, children, and heart failure patients. Models are mainly multidisciplinary and, although staffing and workload may also have an impact on provided care, this was not enough investigated.

**Discussion:**

Community health services described in European literature in the last decade are in line with population needs and suggest different suitable models and settings according to different care needs. Community care should be strengthened in health systems, although the influence of staffing, workload, and work environment on nursing care should be investigated by developing new management models.

**Conclusions and implications for health policy:**

Community care models are heterogeneous across Europe, and the optimum organizational structure is not clear yet. Future policies should consider the impact of community care on both health and economic outcomes and enhance nursing contributions to care.

## INTRODUCTION

In the last two decades, there have been epidemiological changes, pharmaceutical and technological progress, financial restraints, and a lack of human resources all over the world. This has led to an increased incidence of noncommunicable diseases, longer life expectancy and years lived with disability, higher incidence of frailty (OECD, 2021; WHO, 2022a, 2022c; McKee & Healy, 2001), and a reduction in in‐hospital length of stay (McKee & Healy, 2001; OECD, 2021). Noncommunicable diseases need special and multi‐professional attention (OECD, 2021; WHO, 2022c, 2023), often after the patient's discharge from the hospital (Coffey et al., 2019; Veenstra & Gautun, 2021).

Most of a population's needs could be effectively managed in community care settings by ensuring the availability of good‐quality healthcare services and adequately equipped facilities, such as general practice, family nursing, nursing homes, community hospitals, district care, continuity of care units, and palliative care networks (Davidson et al., 2019; Spasova et al., 2018). Current organizational models for community healthcare services across Europe are manifold, and there are still challenges that need to be overcome regarding care organization and evaluation (Spasova et al., 2018). Besides the lack of standardized care and quality indicators across countries (Joling et al., 2018; Veldhuizen et al., 2021), the main issues in this field include service fragmentation and scarcity (WHO, 2022b). Ideally, community care services should be person‐centered, ensure continuity of care and availability of adequate equipment, and be evaluated over time (Spasova et al., 2018).

Considering some emerging population needs also in the aftermath of the COVID‐19 pandemic, community healthcare services in Italy are undergoing a reorganization. In this process, nurses play a pivotal role in the organization and delivery of care (Ministero della Salute, 2022). Hence, the need to gain insights into this reorganization and evaluate nursing contribution in delivering community care is paramount. This would be ensured by a deep investigation of existing community care in Italy (Bagnasco et al., 2023), as well as features and outcomes of community health services documented in European literature.

Considering that knowledge of in‐hospital optimum organizational models, staffing, and skill mix of healthcare professionals has been fundamental to a better understanding of care outcomes (Aiken et al., 2017), this review aimed to describe the organizational models of community healthcare documented in European literature, their outcomes on service users, the contribution of nursing to care, and nursing‐related determinants of those outcomes.

Based on the available European literature, this systematic review addressed the following questions: (i) What are the main features of community healthcare services documented in European literature in the last decade? (ii) What are the outcomes of these services? (iii) What is the contribution of nursing to care in these services? (iv) What is the role of nursing‐related determinants on community care service outcomes? By addressing these questions, this study would provide insights into services documented in European literature and their outcomes in the last decade, to inform future policies and research.

## METHODS

### Study design

The Italian Centre of Excellence for Research and Nursing Development (CERSI) conducted a systematic literature review based on recommendations of the Joanna Briggs Institute (JBI) Manual for Evidence Synthesis (Aromataris & Munn, 2020; Tufanaru et al., 2020), providing a report by following the Preferred Reporting Items for Systematic Reviews and Meta‐Analyses (PRISMA) checklist (Page et al., 2021).

### Sources of information and search strategy

PICO (population, comparison, intervention, and outcome) was applied to conduct the electronic search strategy:

- Population: patients receiving community health services.
- Intervention: community health services where nurses operate either independently or as part of a multidisciplinary team. These services included primary care, district nursing, community nursing, family nursing, continuity of care, transitional care, home care, community hospital, palliative home care, and school health services. Intervention also accounted for nursing‐related factors that could influence patient outcomes, i.e., staffing levels, nurse‐to‐patient ratios, skill and qualification mix, workload, caseload, and care models.
- Comparison: any type of health services provided as an alternative to the investigated community health service, including usual care. Additionally, intra‐group comparisons were considered, i.e., outcome indicators changing after implementing the service detailed in the intervention. As an inclusive strategy, this component of the PICO was not included in search strings to ensure including studies without a control group.
- Outcomes: quality indicators linked with nursing interventions or care models within community health services, i.e., unplanned rehospitalization, access to emergency medical services, missed care, self‐care, empowerment, self‐efficacy, therapeutic adherence, health literacy, and patient satisfaction.

To individuate community health services denominations and relevant quality indicators (outcomes), a discussion was undertaken among the authors, after independently consulting literature on this topic. Afterward, a preliminary search was performed on PubMed to retrieve search terms. On October 27, 2022, PubMed, CINAHL, Scopus, and Embase databases were searched for relevant studies conducted in the last 10 years (Supplementary Tables S1 and Table S2). Considering the broad coverage provided by these databases, no additional searches were done, which was contrary to the review protocol published in PROSPERO. Since study selection and data extraction lasted almost one year, on October 18, 2023, search strings were relaunched and studies published after October 27, 2022, were examined. The retrieved literature was managed using EndNote X7.8 (Thomson Reuters, New York). Where necessary, university libraries or paper authors were contacted to retrieve full texts.

### Eligibility and inclusion criteria

The following inclusion criteria were considered both at the eligibility (title and abstract analysis) and inclusion (full‐text examination) stages: quantitative original studies, published in peer‐reviewed journals, conducted in European countries, published in English or Italian, examining the impact of community care models including the contribution of nursing to patient outcomes described in the PICO. Hence, all the considered community care included health services. The description of nursing‐related factors influencing patient outcomes was not utilized as an inclusion criterion. Instead, these factors were summarized solely for studies reporting them. References with no abstract or full text available at the eligibility or inclusion stage were excluded.

### Study selection, data extraction, and analysis

Two researchers independently examined titles, abstracts, and eligible full texts. They also extracted data using a standardized and piloted Microsoft Excel® template. This template included author, year of publication, country, research design, population, sample size, sample characteristics, study purpose, intervention(s), comparison (if any), outcome(s), measurement tools, nursing contribution to the model, nursing‐related determinants of outcomes, statistical methods, measures of association, and covariates. Nursing contribution was considered as part of a “community care” model, i.e., nurses working independently or in a multidisciplinary team (Ministero della Salute, 2022). Nursing‐related determinants of patient outcomes (staffing level, nurse‐to‐patient ratio, skill mix, qualification mix, workload, caseload, and care model) were considered as an integral part of community nursing (Alghamdi, 2016; Butler et al., 2019; Cunningham et al., 2019; Ministero della Salute, 2021; Veldhuizen et al., 2021). Moreover, to provide a framework regarding summarized research, the economical features (i.e., whether public or private) of documented interventions were extracted, if reported by authors. For data extraction, disagreements were resolved through discussion with a third researcher.

When summarizing results, studies that reported similar services were clustered and described together under a distinctive label, closely resembling labels identified by the authors of the included manuscripts. The label was shaped by considering the settings for service provision and the main care needs of the patients, regardless of their underlying pathology. Services performed remotely using technology were included in a separate category. This approach was implemented to ensure summarizing together features and outcomes of similar interventions. Due to data heterogeneity, meta‐analyses were not feasible. Consequently, general information of studies, types of community care services and features (setting, population, main purpose of the service, and whether the care model was multidisciplinary), outcomes on service users, the contribution of nursing to care, and nursing‐related determinants of those outcomes, were described using tables and the narrative form. Results were described and critically discussed according to study design, type of comparison performed, and studies’ methodological quality. Each study was assigned an ID (Results section; Supplementary Table S3), which is cited when describing results.

### Methodological quality

Two researchers independently evaluated the methodological quality of the included studies using the JBI critical appraisal tools (Aromataris & Munn, 2020). Disagreements were resolved through discussion with a third researcher. Considering the explorative nature of the review, methodological quality was not considered as an exclusion criterion; hence, low‐quality studies were not excluded.

### Ethical approval, informed consent, and registration

Ethical approval and informed consent were not applicable. The systematic review protocol was registered in PROSPERO (CRD42022383856) on December 25, 2022.

## RESULTS

**FIGURE 1:**
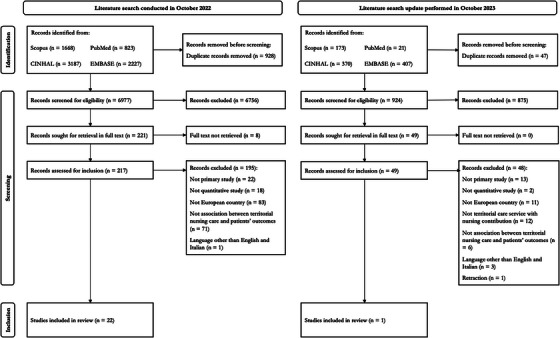
PRISMA flow diagram.

Overall, 8880 references were retrieved, and 7901 of them were examined after removing duplicates. A total of 266 full texts were examined and 23 studies were included (Augestad et al., 2020—A01; Bertelsen et al., 2017—A02; Campbell et al., 2015—A03; Carter‐Stephens, 2020—A04; de Stampa et al., 2014—A05; Facultad & Lee, 2019—A06; Ferrara et al., 2015—A07; Fournaise et al., 2023—A08; González‐Franco et al., 2022—A09; Karlsson et al., 2013—A10 and A11; McGloin et al., 2020—A12; Ng et al., 2014—A13; Phelan et al., 2018—A14; Profili et al., 2017—A15; Röhricht et al., 2017—A16; Senek et al., 2020—A17; Smits et al., 2020—A18; Vainieri et al., 2018—A19; Vianello et al., 2016—A20; Villani et al., 2014—A21; Zimmermann et al., 2016—A22; Zúñiga et al., 2015—A23) (Figure 1).

### Characteristics of included studies

As given in Supplementary Table S3, included studies were conducted in the United Kingdom (*n*  = 6, 26.1%) (A03, A04, A06, A13, A16, and A17), Italy (*n* = 5, 21.7%) (A07, A15, and A19–A21), Spain (A09, A11), Ireland (A12 and A14), Germany (A08 and A22) (*n* = 2, 8.7% each), and other European countries (*n* = 6, 26.1%) (A01, A02, A05, A10, A18, and A23). Years of publication ranged from 2013 to 2023.

Eleven studies (43.5%) were observational (A04, A07, A09, A10, A13–A17, A19, and A23), seven (30.4%) were experimental (A01–03, A08, and A20–22), three (13.0%) were quasi‐experimental (A05, A11, and A18), two (9.7%) were descriptive (A06 and A19), and one (4.3%) was mixed‐method (A12). Multicentric studies were less than half of the total included studies (*n* = 11, 47.8%) (A02, A03, A08–A11, A16, A17, A20, and A23). Sampling was consecutive (*n* = 6, 26.1%) (A05, A10–A12, A20, and A22), random (*n* = 5, 21.7%) (A02, A03, A19, A21, and A23), included the whole reference population (*n* = 5, 21.7%) (A06–A09, A18), convenient (*n* = 1, 4.4%) (A16), purposive (n = 1, 4.4%) (A14), or unclear (*n* = 5, 21.7%) (A01, A04, A13, A15, and A17).

Most of the studies included people with different diseases and characteristics (*n* = 20, 87.0%, total = 44 269 persons) (A01–A16, A18–A22), while three studies (13.0%) (A14, A17, and A23) included healthcare professionals (total = 6332). The mean number of participants was 2213.5 (SD = 4985.4, minimum = 33, maximum = 16 972) and 2110.7 (SD = 2037.2, minimum = 283, maximum = 4307) for users and professionals, respectively.

### Types of community care services and related outcomes

Six types of community care services were identified (Table 1), namely, home care (*n* = 8, 34.8%) (A05–A11, and A13), remote health (*n* = 5, 21.7%) (A01, A03, A12, A20, and A21), community health (*n* = 4, 17.4%) (A04, A14, A17, and A19), primary healthcare (*n* = 4, 17.4%) (A15, A16, A18, and A22), nursing homes (A23), and transitional care (A02) (*n* = 1, 4.5% both). All community services provided healthcare, although they were heterogeneous in terms of the target population, interventions, and organizational characteristics. Moreover, they were delivered at home (e.g., home care), in institutions (e.g., nursing homes), or through facilities and devices, with professionals (e.g., community health, primary healthcare, remote health, and transitional care) acting as a bridge between home setting and institutions. A detailed description of provided services and interventions is reported in Supplementary Table S4, while detailed and summarized descriptions of outcomes are reported in Supplementary Table S5 and Table 2, respectively.

**TABLE 1:** Type and general characteristics of community care services and number of studies including these services.

Service	No. of studies	Characteristics	Financial model (ID)
Home care	8	Encompass a comprehensive and personalized range of health care interventions provided by multidisciplinary teams to patients in their own homes. These services are generally provided to face chronic health issues, patients’ frailty, or special health needs.	Public (A08, A05, A07, A10, A11, and A13) Not reported (A06 and A09)
Remote health	5	Health services delivered by healthcare professionals through electronic communication technologies. These services bridge the gap between geographically distant healthcare professionals and patients, enabling regular monitoring, assessment, and management of conditions, ensuring timely interventions and support, while promoting convenience, accessibility, patient engagement, self‐care, and overall satisfaction with the healthcare experience.	Not reported (A01, A03, A12, A20, and A21)
Community health	4	Integrated and accessible healthcare services provided within local communities (e.g., clinics, districts, ambulatories), encompassing preventive care, patient education, and support to enhance health outcomes, satisfaction, and self‐care.	Public (A14 and A17) Not reported (A04 and A19)
Primary health care	4	Comprehensive and person‐centered healthcare services, generally coordinated or supervised by general practitioners, encompass a multidisciplinary approach, and are delivered in clinics or at patients' homes, ensuring holistic care aimed at promoting self‐care, satisfaction, and reduce access to emergency services.	Public (A15) Not reported (A16, A18, and A22)
Nursing homes	1	Are residential facilities that offer a range of services for individuals requiring long‐term care, short‐term stays, adult day care, and post‐acute care, providing a dedicated living environment for individuals with chronic or terminally ill conditions. With a focus on multidisciplinary interventions, including rehabilitation and nursing care, nursing homes strive to meet the needs of residents, enhance their quality of life, and ensure their well‐being in a residential setting, catering to both their ongoing care and support requirements.	Mixed^a^ (A23)
Transitional care	1	Encompass a coordinated and multidisciplinary approach to care, specifically targeting patients recently discharged from the hospital after experiencing acute health problems. These services aim to facilitate a seamless transition from the hospital to home or community settings by offering a range of interventions, including health education, physical exercise, smoking cessation support, dietary advice, clinical evaluations, medical visits, with the ultimate objective of enhancing patients' treatment adherence and optimizing their overall health outcomes throughout the transitional period.	Public (A02)

^a^ Mixed: Nursing homes financed with an approximately equal proportion of public, private, or privately subsidized (operating under private law but guaranteed by the public sector) systems.

**TABLE 2:** Studies results according to the care model and study design.

Outcome		Home care	Remote health	Community health	Primary health	Nursing homes	Transitional care
Hard	Access to emergency	Quasi‐experimental + Observational +	Randomized controlled trial −	Observational +	Observational ‐	–	–
	Repeated hospitalization	Randomized controlled trial + Quasi‐experimental + Observational +	Randomized controlled trial +	–	–	–	–
	Hospital admission due to HF	Observational +	–	–	–	–	–
	Unplanned hospital admission	Quasi‐experimental +	–	–	–	–	–
PROs	Adherence to treatment	–	Randomized Controlled trial +	–	–	–	Randomized controlled trial +
	Empowerment	–	Mixed‐method +	–	–	–	–
	Patient satisfaction	Observational + Cross‐sectional + + Descriptive +	Randomized Controlled trial − Mixed‐method +	Descriptive −	Quasi‐experimental + Observational +	–	–
	Self‐efficacy	–	–	–	Randomized controlled trial +	–	–
	Self‐care	–	Randomized controlled trial −	Descriptive −	–	–	–
NROs	Missed nursing care associated with staffing	–	–	Cross‐sectional + +	–	Cross‐sectional +	–

Abbreviations: NROs: nurses reported outcomes; PROs: patients reported outcomes.

*Note*: Each + indicates a study demonstrating improved outcome pre‐post or compared with control.

Each − indicates a study demonstrating no outcome differences pre‐post or compared with control.

#### Home care services

Home care services were provided at home by multidisciplinary teams for early identification of home care clients at risk of hospitalization (A08) and to manage frailty due to aging (A05, A07, and A10), long‐term conditions (e.g., heart failure and diabetes) (A09 and A11), or special health needs of children and critically ill patients (A06 and A13). Multidisciplinary interventions consisted of comprehensive clinical assessment and care planning (A05 and A11), monitoring and follow‐up (A05, A06, A08–A11, and A13), patients’ stabilization at home (A06, A09, A11, and A13), education and self‐care promotion (A09, A11, and A13), and comprehensive clinical and social services (A07).

Nursing interventions included assessment and care planning (A05), home visits and consultations (A09, A10), supervision and educational activities (A13), care coordination (A05), and decision‐making (A08), although they were not always fully described.

These services were largely provided to older people. In a quasi‐experimental study, authors compared 105 very frail older people, who were cared for by a two‐person case manager team (i.e., a nurse and a physician) supported by a geriatrician, and 323 similar persons receiving usual care. The experimental group showed lower unplanned hospital admissions after one year (adjusted OR = 0.39, 95%CI = 0.16–0.98) (A05). Also, in a large randomized controlled trial (RCT) involving 2464 older people and in which a prediction algorithm was applied by nurses to prevent hospital readmission, the intervention group showed a significantly lower incidence of readmissions compared with the usual care group (incident rate ratio IRR = 0.41, 95%CI = 0.24–0.68, *p* = 0.0007) (A08). Finally, a quasi‐experimental study involved 101 older people affected by long‐term conditions who were cared for through a multidisciplinary integrated care pathway comprising communication, care coordination, and home‐based care, instead of usual care (A11). Results highlighted a significant reduction in the average number of accesses to emergency services (*p* ≤ 0.001) and repeated hospitalizations (*p* = 0.008).

Similar results were obtained in an observational study of 809 patients with heart failure, who were included in an intensive and protocolized follow‐up program involving a comprehensive clinical approach, an in‐person and telephone follow‐up, educational interventions, and care in case of decompensation. A significantly lower risk of rehospitalizations due to heart failure was documented (*p* < 0.001) compared with usual care patients (*n* = 2053) (A09). Moreover, 33 acutely ill children were observed after being managed at home by a nursing matron who may consult with a pediatrician. A 17.3% reduction in repeated hospitalization and a 5% reduction in access to emergency services were observed, although no statistical tests were performed in this observational study (A13).

Another highly investigated outcome was user satisfaction. Among 417 older people included in a cross‐sectional study to observe the outcome of the provision of home clinical and social services, 67.2% declared their trust in the model, with higher percentages among city residents (72.1%) compared with people living in peripheral areas (43.0%) (A07). Although overall user satisfaction is highly valued, these services seem to be more appreciated by older people who receive them at home rather than in special accommodations (*p* = 0.004), as revealed in a cross‐sectional study involving 166 older persons who received at least two home/accommodation visits by home care nurses (A10).

Finally, in other populations, satisfaction was high in 206 persons with various acute ill who received intensive care treatments at home (99.0% satisfied or very satisfied) (A06) and in 33 acutely ill children managed at home by a nursing matron who may consult with a pediatrician (94% described as excellent the care received and 6% as good) (A13).

#### Remote health services

These services were delivered by healthcare professionals using digital communication technologies to people affected by a variety of surgical and medical conditions (e.g., postsurgical stoma, heart failure, diabetes, and COPD) (A01, A03, A12, A20, and A21).

Vital signs and other clinical data were daily transmitted to multidisciplinary professionals (mainly physicians or nurses) who decided either to modify therapy, plan a nurse visit at home, or refer patients to specialists, including psychologists (A20 and A21). Main nursing interventions were follow‐up through teleconsultation (A01, A03, and A12), remote monitoring of clinical conditions (A12, A20, and A21), and arranging appointments with medical specialists (A01 and A03). Other nursing activities included providing self‐care recommendations (A03), collecting clients’ requests (A21), performing clinical examinations (A01), and home visits (A20).

One RCT evaluated the effects of multidisciplinary telemonitoring on repeated hospitalizations and access to emergency in a sample of 230 persons with COPD. Results showed a positive impact of the service on repeated hospitalization (IRR usual care vs. intervention group = 0.46, 95% CI = 0.24–0.89) as opposed to access to emergency (IRR usual care vs. intervention group = 0.94, 95% CI = 0.76–1.18) (A20).

Compared with 3‐monthly in‐person visits, multidisciplinary telemonitoring showed better compliance to treatment in a sample of 80 patients with chronic heart failure (intervention group = 3.8 ± 0.5 vs. control group = 3.0 ± 0.8; *p* < 0.05) (A21). Also, empowerment significantly improved in a sample of 39 patients affected by type II diabetes after including telemonitoring, led by a clinical nurse specialist, to usual care (intervention group pre vs. post at 3 months, mean difference = +0.72, *p* < 0.001) (A12). Compared with traditional hospital nurse–led follow‐up, teleconsultation performed by stoma nurses did not impact differently on self‐care in a sample of 110 persons with postsurgical stoma (*p* = 0.825) (A01).

Finally, client satisfaction was high (i.e., scored above 4 out of 5) for telemonitoring performed by a clinical nurse specialist on 39 patients with type II diabetes (A12), although another study involving 110 people with postsurgical stoma in an RCT found no differences between nursing teleconsultation and usual care in a hospital setting (good overall experience in the intervention group *n* = 103, 49.9% vs. control group *n* = 149, 59.1%; *p* = 1.000) (A01). Also, in a trial including 16 211 persons in primary care, general practitioner–led telephone triage and usual care showed higher user satisfaction compared with nurse‐led computer‐supported telephone triage (mean difference: + 3.94, 95% CI = 1.88–5.99 and 2.60, 95% CI = 0.58–4.63, respectively) (A03).

#### Community health services

Community health services were delivered through community clinics, districts, outpatient services, or directly at home, if necessary (A04, A14, A17, and A19), to manage long‐term conditions, such as diabetes and heart failure (A19), or to address the healthcare needs of people receiving enteral tube feeding at home (A04). These services included nursing care (A04, A14, A17, and A19), physiotherapy, dietetic recommendations, and medical specialist visits (A19). Where described, nursing interventions consisted of providing educational activities and support (A04 and A19), scheduling specialist visits and follow‐ups (A19), and managing complications (A04).

In an observational study involving an unknown number of people receiving nurse‐led enteral tube feeding, the authors reported a considerable reduction (−93%) in the number of accesses to emergency services, which translated to better user experience (A04). In a descriptive study involving 1300 patients with single and multiple long‐term conditions, a chronic care model, compared with standard care, did not influence patient satisfaction (people with single long‐term conditions OR = 0.91, *p* = 0.63; people with multiple long‐term conditions OR = 1.09, *p* = 0.61) or self‐care (people with single long‐term conditions OR = 1.88, *p* = 0.23; people with multiple long‐term conditions OR = 0.53, *p* = 0.12). The model was introduced in community services and applied by a multidisciplinary team (GP, nurses, physiotherapists, dieticians, and medical specialists). The authors documented that during the observational period of the project, changes occurred in the GPs' practice based on the model itself (A19).

Finally, two cross‐sectional studies involving 283 and 1742 community/public health nurses documented a significant association between organizational factors or staffing and missed care (A14) and an increased prevalence of missed nursing care in understaffed services (A17). Younger community nurses were more likely to miss several direct and indirect nursing activities (*p* < 0.05) and those without a bachelor's degree were more likely to miss “report writing” (*p* < 0.05). Moreover, detected differences in missed nursing care between adequately staffed and understaffed services ranged from +5.1% to +15.5% (A17).

#### Primary healthcare services

Primary healthcare included multidisciplinary services coordinated or supervised by general practitioners (A15, A16, A18, and A22). These services were generally delivered, in clinics or at home (if required), to persons affected by mental disorders (A16, A22), long‐term conditions (A15), or low‐complexity health problems (A18).

Due to the diverse target populations that require these services, multidisciplinary and nursing interventions were heterogeneous. Educational programs (A15 and A16), activities promoting self‐management of disease (A15, A22), care planning (A15, A16), as well as monitoring and follow‐up (A15, A16, A22), were the most provided multidisciplinary interventions. Nurses were responsible for measuring vital signs and planning routine services (A15), administering medications, scheduling lifestyle services, educating patients (A16), and visiting them at home (A18).

A chronic care model for 16 972 type II diabetic patients comprising multiprofessional intervention, active patient involvement in the educational program, personalized evidence‐based care, and scheduled follow‐up showed no differences in accessing emergency services (IRR = 0.94, 95% CI = 0.84–1.01) compared to traditional care based on the essential level of assistance (A15). In an RCT involving 325 patients with psychiatric disorders, a primary care‐based complex intervention to promote self‐management and enhance self‐efficacy demonstrated a significant increase in patient self‐efficacy when compared with usual care led by general practitioners (*p* = 0.004) (A22).

Finally, clients with low‐complexity health problems showed higher satisfaction (*n* = 217) when receiving evening and weekend home visits by trained nurses compared with 412 clients receiving visits only from their general practitioners (8.6/10, 95%CI = 8.5–8.8; vs. 8.3/10 95%CI = 8.2–8.4) (A18). Also, 126 psychiatric patients involved in an enhanced primary care pathway based on a multidisciplinary collaboration between healthcare providers and educational activities reported high satisfaction (from 8.8/10 to 9.4/10) (A16).

#### Nursing homes

Nursing homes were investigated only in one cross‐sectional study including 4307 direct care workers employed in 156 nursing homes in Switzerland. They included a variety of residential services offering long‐term care, short stays, adult day care, and post‐acute care (A23). Nursing homes were accessed mainly by people with dementia, older people with psychiatric disorders, or persons needing palliative care. Even if few details were reported in the study, these services were based on multidisciplinary interventions, such as rehabilitation and nursing care. Results showed a significant relationship between the adequacy of staffing levels, workload, and a positive healthcare environment with implicit rationing of nursing care (*p* < 0.05) (A23).

#### Transitional care services

Transitional care services were assessed in one RCT conducted in Denmark on a sample of 212 patients exposed to shared care cardiac rehabilitation after acute coronary syndrome. The services involved a set of multidisciplinary interventions to ensure continual care for patients discharged from the hospital and included health education, physical exercise, abstinence from smoking, dietary advice, clinical evaluation, and medical visits (A02). Specifically, they were provided after hospitalization due to acute coronary syndrome in community services or in hospital outpatient clinics to improve adherence to treatment. Compared to patients exposed to a hospital cardiac‐based rehabilitation program, no significant difference in outcome was reported (RR = 0.98, 95% CI = 0.73–1.32) (A02).

### Nursing determinants

The most frequently reported nursing‐related determinant of outcomes was skill mix (*n* = 7, 30.4%) (A01, A02, A04, A05, A07, A12, and A22), followed by staffing models (*n* = 6, 26.1%) (A01, A04, A06, A07, A22, and A23), type of contract (*n* = 3, 13.0%) (A16, A14, and A22), caseload (*n* = 3, 13.0%) (A14, A22, and A23), and nurse‐to‐patient ratio (*n* = 2, 8.7%) (A05, A22). Workload was not always reported in the included studies, but when it was, the authors did not always describe its association with patient outcomes (A14 and A17) (Supplementary Table S6).

### Methodological quality

Methodological quality was rated from low to high in the included studies, with high variability among studies according to the study design. The main concerns were related to treatment concealment and blinding of patients, providers, and outcome assessors in RCTs; assessment of confounding factors and handling of incomplete follow‐up in quasi‐experimental studies; and standardization of outcome assessment and handling of confounding factors in cross‐sectional studies (Supplementary Table S7).

## DISCUSSION

Considering the lessons learned from the COVID‐19 pandemic and the need to improve the quality of community care services, we performed this review to summarize the organizational models of community healthcare documented in European literature, their outcomes on service users, the contribution of nursing to care, and nursing‐related determinants of outcomes reported. The ultimate goal of this review was to contribute to the reorganization of community services in Italy (Ministero della Salute, 2022) and inform future European policies.

The results highlight a stable scientific production on the topic over the last decade, except for the year 2020 (the COVID‐19 year), when more than one‐quarter (26.1%) of included studies were published, perhaps pushed by the need to find innovative and more resilient models to provide community care. Studies were conducted mainly in the United Kingdom and Italy, with the rest conducted in other European countries, highlighting the need for further studies in various contexts.

Almost half of the studies were multicentric, ensuring good representativeness of involved contexts. Finally, despite the limited number of experimental studies, only a quite small number of included studies utilized unclear or high‐risk sampling methods (30.4%). Nevertheless, the results of this review should be interpreted and generalized with caution since some observational studies showed methodological weaknesses, especially regarding the management of confounding factors. As a further limitation, it should be considered that evaluated outcomes do not encompass all those that may be influenced by community care services. To gather a comprehensive overview of the impact of these services on outcomes, quantitative findings should be integrated with qualitative research involving stakeholders, as this can highlight relevant aspects or factors. Examples of such factors include team dynamics, features of team–patient relationship, potential health system barriers, patient caseload, and attitudes of team members (Lalani et al., 2019; Leask et al., 2020). Lastly, study heterogeneity did not allow meta‐analysis of data. Instead, the strengths of this review include rigorous methodology based on international standards, which ensured internal consistency, reproducibility, and accuracy of results.

Community care documented in European literature tends to be very complex and heterogeneous. Different types of services were described, although these services could be clustered considering the provision setting and main needs of patients accessing them.

Almost all studies reporting financial coverage details (Table 1, *n* = 10) were free of charge for patients. Even if not explicitly stated, it is reasonable to assume that in the other included studies, services were also free of charge as they were provided within the context of experimental research and/or in countries known for their universal healthcare coverage. This made it difficult to assess differences according to contextual factors, such as economic coverage (public or private), regarding outcomes. Hence, it is advisable that policymakers develop services informed by the results of this review, local needs, and available resources. Moreover, policymakers should endorse additional research on health and economic outcomes of services addressing predominantly emerging needs within their local context, fostering a direct and reciprocal exchange between research and practice.

In line with emerging population needs (OECD, 2021; WHO, 2022a, 2022c) and technological advancements, most of the investigated services were home care and remote health. Moreover, almost all services were multidisciplinary, though including different activities and tasks performed by nurses, such as assessment and care planning, home visits and consultations, support and education, care coordination, continuous follow‐up, administration of medications, and monitoring of clinical conditions. Most of the studies refer to very specific types of patients and each type of service often refers to the same type of patients. For instance, home care services were mainly explored in regard to older people, remote services mainly targeted patients with specific diseases, such as heart failure or COPD, and primary care services mainly considered low‐complexity patients. Outcomes included “hard” indicators (i.e., access to emergency services, repeated hospitalization, hospital readmissions due to heart failure, and unplanned hospital admission) and “soft” indicators reported by either users (i.e., satisfaction, therapeutic adherence, empowerment, self‐efficacy, and self‐care) or professionals (i.e., missed nursing care). The impact of community care on access to emergency services, satisfaction, and adherence to treatment remains unclear. Community care with significant nursing contribution reduced rehospitalization of people with long‐term conditions, frail or older persons, children, and patients with heart failure. Through different delivery types and modalities, appropriate community care prevents unnecessary access to and treatment in hospitals (MacNeill et al., 2021). Considering the pivotal role of clinical surveillance and early interventions, patient and caregiver education is fundamental as patients and caregivers are required to have high autonomy and self‐care abilities to manage a disease.

Factors confirmed to reduce repeated hospitalization in community care settings include continuous follow‐up and monitoring (Masotta et al, 2024). Other factors that appear to reduce this outcome are home visits and clinical stabilization at home, education on self‐care, and empowerment programs. An additional strategy not delineated in the included studies, but which has demonstrated the potential to enhance both public health management and clinical outcomes, is nursing prescription (Laurant et al., 2018; Maier, 2019; Weeks et al., 2016). In this context, the World Health Organization advocates for the adoption of strategies that promote collaboration among healthcare professionals to address prescription requirements (WHO, 2020). This approach may enable nurses to fully utilize their professional capabilities while optimizing both patient and system outcomes (WHO, 2020).

Similar to in‐hospital settings (Chiappinotto et al., 2022; Imam et al., 2023), missed nursing care seems to be associated with nurse staffing, workload, and work environment, although this relationship has not been investigated adequately.

### Implications for nursing and health policy

Community care with significant nursing contribution should be largely boosted across Europe, while investigating its outcomes, in order to individuate the optimum organizational structure in each context and for each target population. In fact, available data suggest that each target population may be effectively cared for in specific settings and through organized ad hoc services.

Organizational and nursing determinants should be accurately considered and investigated when developing community services, considering the impact of multidisciplinary and nursing interventions on population outcomes.

## CONCLUSIONS

In the last decade, research on community care in Europe has been undertaken through complex and heterogeneous services targeting different types of patients, and by evaluating heterogeneous outcomes. In this complex context, the following main insights have been revealed:

- Community services are organized according to a specific type of setting, population needs, or technology used.
- Target populations considered in the literature are in line with worldwide epidemiology and worldwide unmet population needs.
- Community services can be multidisciplinary or delivered by one professional.
- Community services have a positive effect on rehospitalization, while conflicting results were retrieved for other outcomes.
- Staffing, workload, and work environment of community services appear to influence nursing care, although this relationship is not sufficiently investigated in the literature.

Future research should include both hard and soft outcomes and investigate their relationship with the organizational determinants of care. In addition, an improvement in the methodological quality of studies is recommended.

## AUTHOR CONTRIBUTIONS

*Study design*: Angelo Dante, Laura Rasero, Loredana Sasso, Annamaria Bagnasco, Rosaria Alvaro, Duilio Fiorenzo Manara, Gennaro Rocco, Maurizio Zega, Giancarlo Cicolini, Beatrice Mazzoleni, and Loreto Lancia. *Data collection*: Valeria Caponnetto, Angelo Dante, Khadija El Aoufy, Maria Ramona Melis, Giulia Ottonello, Francesca Napolitano, Fabio Ferraiuolo, Francesco Camero, Angela Cuoco, and Ilaria Erba. *Data analysis*: Valeria Caponnetto and Angelo Dante. *Study supervision*: Angelo Dante, Laura Rasero, Loredana Sasso, Annamaria Bagnasco, Rosaria Alvaro, Duilio Fiorenzo Manara, Gennaro Rocco, Maurizio Zega, Giancarlo Cicolini, Beatrice Mazzoleni, and Loreto Lancia. *Manuscript writing*: Valeria Caponnetto, Angelo Dante, Khadija El Aoufy, Maria Ramona Melis, Giulia Ottonello, Francesca Napolitano, Fabio Ferraiuolo, Francesco Camero, Angela Cuoco, and Ilaria Erba. *Critical revisions for important intellectual content*: Angelo Dante, Valeria Caponnetto, Laura Rasero, Loredana Sasso, Annamaria Bagnasco, Rosaria Alvaro, Duilio Fiorenzo Manara, Gennaro Rocco, Maurizio Zega, Giancarlo Cicolini, Beatrice Mazzoleni, and Loreto Lancia.

## CONFLICT OF INTEREST STATEMENT

The authors declare that they have no known competing financial interests or personal relationships that could have appeared to influence the work reported in this paper.

## FUNDING INFORMATION

This research did not receive any specific grant from funding agencies in the public, commercial, or not‐for‐profit sectors.

## Supporting information

Supporting information

## Data Availability

The authors make the original data available upon reasonable request.
