# Supplementary material for: Community health services in European literature: A systematic review of their features, outcomes, and nursing contribution to care
Source: Int Nurs Rev. 2024 Jul 29;71(4):716–28. doi: 10.1111/inr.13033 (PMC11600541; doi:10.1111/inr.13033)
Supplement: Supplementary file 1 — Supporting information [file INR-71-716-s001.docx]

**Table S1. List of keywords**

| **Community care services / nursing related determinants** | **Patients’ Outcomes** |
| --- | --- |
| Primary Health Care, District nurs*  Community Health Nursing  Family Nursing  Family Nurse Practitioners  Family Neighborhood nurs*  Community nurs*  Nurses, Community Health  Community care registered nurse*  Public Health Nursing  School Nursing  Occupational Health Nursing  Community Health Services  Continuity of Patient Care  Long-term care  Home Care Services  Home care practice  Home care nurs*  Advanced Home Care Nurs*  Home Health Nursing  Palliative home care  Home-based palliative care  Home palliative care  Hospitals  Community, Intermediate Care Facilities  Transitional care, Nursing Homes  Home nurse service*  Community nurse led care | Repeated Hospitalization*  Patient Readmission  Access Emergency Medical Service*  Emergency Medical Services  Missed care  Unfinished care  Care left undone  Self-care  Empowerment  Self-Efficacy  Patient Compliance  Health literacy  Patient Satisfaction |
| **Nursing-related determinants** |  |
| Staffing model  Staff level  Nurse-to-patient-ratio  Skill mix  Qualification mix  Nursing Staff  Workload  Caseload  Care model*  Models, Nursing |  |

**Table S2. Electronic search strings**

| **PUBMED** |
| --- |
| ("primary health care"[MeSH Terms] OR "district nurs*"[Title/Abstract] OR "community health nursing"[MeSH Terms] OR (("family nursing"[MeSH Terms] OR ("family"[All Fields] AND "nursing"[All Fields]) OR "family nursing"[All Fields]) AND "family nurse practitioners"[MeSH Terms]) OR (("familialities"[All Fields] OR "familiality"[All Fields] OR "familially"[All Fields] OR "familials"[All Fields] OR "familie"[All Fields] OR "family"[MeSH Terms] OR "family"[All Fields] OR "familial"[All Fields] OR "families"[All Fields] OR "family s"[All Fields] OR "familys"[All Fields]) AND "neighborhood nurs*"[Title/Abstract]) OR "community nurs*"[Title/Abstract] OR "nurses, community health"[MeSH Terms] OR (("communal"[All Fields] OR "communalism"[All Fields] OR "communalities"[All Fields] OR "communality"[All Fields] OR "communally"[All Fields] OR "commune"[All Fields] OR "communes"[All Fields] OR "community s"[All Fields] OR "communitys"[All Fields] OR "residence characteristics"[MeSH Terms] OR ("residence"[All Fields] AND "characteristics"[All Fields]) OR "residence characteristics"[All Fields] OR "communities"[All Fields] OR "Community"[All Fields]) AND "care registered nurs*"[Title/Abstract]) OR "public health nursing"[MeSH Terms] OR "occupational health nursing"[MeSH Terms] OR “School Nursing”[MeSH Terms] OR "community health services"[MeSH Terms] OR "continuity of patient care"[MeSH Terms] OR "long term care"[MeSH Terms] OR "home care services"[MeSH Terms] OR "home care practice"[Title/Abstract] OR "home care nurs*"[Title/Abstract] OR (("advance"[All Fields] OR "advanced"[All Fields] OR "advancement"[All Fields] OR "advancements"[All Fields] OR "advances"[All Fields] OR "advancing"[All Fields]) AND "home care nurs*"[Title/Abstract]) OR "home health nursing"[MeSH Terms] OR "palliative home care"[Title/Abstract] OR "home based palliative care"[Title/Abstract] OR "home palliative care"[Title/Abstract] OR "hospitals, community"[MeSH Terms] OR "intermediate care facilities"[MeSH Terms] OR (("transitional care"[MeSH Terms] OR ("transitional"[All Fields] AND "Care"[All Fields]) OR "transitional care"[All Fields]) AND "nursing homes"[MeSH Terms]) OR (("home environment"[MeSH Terms] OR ("Home"[All Fields] AND "environment"[All Fields]) OR "home environment"[All Fields] OR "Home"[All Fields]) AND "nurse service*"[Title/Abstract]) OR (("communal"[All Fields] OR "communalism"[All Fields] OR "communalities"[All Fields] OR "communality"[All Fields] OR "communally"[All Fields] OR "commune"[All Fields] OR "communes"[All Fields] OR "community s"[All Fields] OR "communitys"[All Fields] OR "residence characteristics"[MeSH Terms] OR ("residence"[All Fields] AND "characteristics"[All Fields]) OR "residence characteristics"[All Fields] OR "communities"[All Fields] OR "Community"[All Fields]) AND "nurse led care"[Title/Abstract])) AND ("staffing model"[Title/Abstract] OR "staff level"[Title/Abstract] OR "Nurse-to-patient-ratio"[Title/Abstract] OR "Skill mix"[Title/Abstract] OR "qualification mix"[Title/Abstract] OR "nursing staff"[MeSH Terms] OR "workload"[MeSH Terms] OR "Caseload"[Title/Abstract] OR "care model*"[Title/Abstract] OR "models, nursing"[MeSH Terms]) AND ("repeated hospitalization*"[Title/Abstract] OR "patient readmission"[MeSH Terms] OR (("access"[All Fields] OR "accessed"[All Fields] OR "accesses"[All Fields] OR "accessibilities"[All Fields] OR "accessibility"[All Fields] OR "accessible"[All Fields] OR "accessing"[All Fields]) AND "emergency medical service*"[Title/Abstract]) OR "emergency medical services"[MeSH Terms] OR "missed care"[Title/Abstract] OR "unfinished care"[Title/Abstract] OR "care left undone"[Title/Abstract] OR "self care"[MeSH Terms] OR "empowerment"[MeSH Terms] OR "self efficacy"[MeSH Terms] OR "patient compliance"[MeSH Terms] OR "health literacy"[MeSH Terms] OR "patient satisfaction"[MeSH Terms]) |
| **SCOPUS** |
| ( ( TITLE-ABS-KEY ( "primary health care nurs*" OR "district nurs*" OR "nurs* community health" OR "family nurs*" OR "family nurs* practitioner" OR "nurs* practitioner*" OR "family neighborhood nurs*" OR "community nurs*" OR "community care registered nurse*" OR "community health nurs*" OR "public health nurs*" OR "nurs* public health" OR "school nurs*" OR "occupational health nurs*" OR "community health service*" OR "continuity of care" OR "long-term care" OR "home care service*" OR "home care practice" OR "home care nurs*" OR "advanced home care nurs*" OR "home health care" OR "visiting nurs*" OR "palliative home care" OR "home-based palliative care" OR "home palliative care" OR "community hospital" OR "intermediate care facilit*" OR "transitional care" OR "nursing home*" OR "community dwelling" OR "home nurse service*" OR "home nurs*" OR "community nurse led care" ) ) AND ( TITLE-ABS-KEY ( "staffing model*" OR "staff level*" OR "nurse-to-patient-ratio" OR "skill-mix" OR "qualification mix" OR "staff*" OR "workload" OR "caseload" OR "care model*" ) ) ) AND ( TITLE-ABS-KEY ( "unplanned hospital readmission*" OR "repeated hospitalization*" OR "hospital readmission*" OR "access emergency medical service*" OR "missed care" OR "unfinished care" OR "care left undone" OR "self-care" OR "empowerment" OR "self-efficacy" OR "therapeutic compliance" OR "patient adherence" OR "health literacy" OR "patient satisfaction" ) ) |
| **EMBASE** |
| (('family nurse practitioner'/exp OR 'patient care'/exp OR 'occupational health nurse'/exp OR 'rural health care'/exp OR 'preventive health service'/exp OR 'public health service'/exp OR 'home palliative care':ab,ti OR 'community hospital'/exp OR 'transitional care'/exp OR 'school health nursing'/exp OR 'school health service'/exp OR 'visiting nursing service'/exp OR 'community care'/exp OR 'primary health care'/exp OR 'nurse led care':ab,ti OR 'primary health care nurs*':ab,ti OR 'district nurs*':ab,ti OR 'family nurs*':ab,ti OR 'public health nurs*':ab,ti OR 'community health service':ab,ti OR 'continuity of care':ab,ti) AND ('staffing model':ab,ti OR 'staff level':ab,ti OR 'nurse-to-patient-ratio':ab,ti OR 'skill mix'/exp OR 'qualification mix':ab,ti OR 'nursing staff'/exp OR 'workload'/exp OR 'caseload'/exp OR 'care model':ab,ti OR 'nursing model':ab,ti OR 'nursing theory'/exp)) AND ('unplanned hospital readmission*':ab,ti OR 'repeated hospitalization*':ab,ti OR 'hospital readmission'/exp OR 'health care access'/exp OR 'access emergency medical service':ab,ti OR 'missed care':ab,ti OR 'unfinished care':ab,ti OR 'care left undone':ab,ti OR 'self care'/exp OR 'self concept'/exp OR 'self-efficacy':ab,ti OR 'empowerment'/exp OR 'patient empowerment'/exp OR 'therapeutic compliance':ab,ti OR 'patient compliance'/exp OR 'treatment refusal'/exp OR 'patient non-compliance':ab,ti OR 'patient adherence':ab,ti OR 'patient non adherence':ab,ti OR 'health literacy'/exp OR 'patient satisfaction'/exp) |
| **CINHAL** |
| TX ( “primary healthcare nurs*” OR “district nurs*” OR “family nurs*” OR “neighborhood nurs*” OR “community nurs*” OR “public health nurs*” OR “community health nurs*” OR “school nurs*” OR “school health nurs*” OR “occupational health nurs*” OR “community health services OR “continuity of care” OR “long term care” OR “home care services” OR “home health care” OR “home nurs*” OR “home care practice” OR “visiting nurs*” OR “home health services” OR “palliative home care” OR “home-based palliative care” OR “community hospital” OR “Intermediate care facilities” OR “transitional care” OR “nurs* led care” ) AND TX ( “staffing model” OR “staff level” OR “nurse-to-patient-ratio” OR “skill mix” OR “nursing staff” OR “workload” OR “caseload” OR “care model” OR “nursing model” ) AND TX ( “unplanned hospital readmission” OR “repeated hospitalization” OR “hospital readmission” OR “readmission” OR “re-hospitalization” OR “access emergency medical service*” OR “missed care” OR “unfinished care” OR “care left undone” OR “self-care” OR “self-efficacy” OR “empowerment” OR “therapeutic compliance” OR “patient adherence” OR “patient satisfaction” OR “health literacy” ) |

Table S3. Characteristics of included studies

| **Study** | **ID** | **Country** | **Study Aim(s)** | **Design** | **Multicentric** | **Population** | **Sampling** | **Sample** | **Comorbidity** |
| --- | --- | --- | --- | --- | --- | --- | --- | --- | --- |
| Augestad et al. 2020 | A01 | NO | To obtain insight into the quality of life of patients with a stoma, followed up in a hospital outpatient setting (controls) or by teleconsultation (intervention). Healthcare resource use, organization and patient satisfaction with the health service provided was assessed. | RCT | No | People with postsurgical stoma (ileostomy or colostomy) | Not Specified | 110 | No |
| Bertelsen et al. 2017 | A02 | DK | To establish a phase II model of Shared Care-Cardiac Rehabilitation (SC-CR) between hospitals, general practitioners, and municipalities and to compare adherence and efficacy concerning risk factor management and pharmacological treatment in SC-CR with hospital based-Cardiac Rehabilitation (H-CR) after acute coronary syndrome in a randomized controlled trial. | RCT | Yes | People hospitalized due to ACS | Randomized | 212 | Yes |
| Campbell et al. 2015 | A03 | UK | To assess the impact of GP-led telephone triage and nurse-led computer-supported telephone triage, in comparison with usual care, on primary care workload and cost, patient experience of care, and patient safety and health status for patients requesting same-day consultations in general practice. | RCT | Yes | Primary care users | Randomized | 16211 | No |
| Carter-Stephens 2020 | A04 | UK | To prevent unplanned hospital attendances and improve patients’ overall experience of HETF, with individualized training, support and dedicated specialist staff | Obs | No | People receiving enteral tube feeding in community clinic or at home if necessary | Not Specified | 100 | No |
| de Stampa et al. 2014 | A05 | FR | To evaluate the impact on hospital admissions of the ‘CO-ordination Personnes Age’ model, which provides integrated primary care with intensive case management for community-dwelling, very frail older people | Q-E | No | Very frail older people | Consecutive | 428 | No |
| Facultad et al. 2019 | A06 | UK | To report on an evaluation of the GSTT@home service with respect to patient satisfaction. | D | No | People with various acute ill, need intensive care treatments | Whole population | 206 | No |
| Ferrara et al. 2015 | A07 | IT | To assess the quality of life of the older person, considered in the specific context of the home context, and to provide a concise collection of data useful for understanding and quantifying the phenomenon and for developing health and social policies based on objective information and indicators. To measure the degree of user satisfaction with the quality of the health care received and the services provided by the home care centre. | C-S | No | Older people | Whole population | 500 | No |
| Fournaise et al. 2023 | A08 | DE | To evaluate the efficacy of the Prevention of Acute Admission Algorithm (PATINA), a prediction model and a decision support tool, in preventing hospital admission of community-dwelling older adults and the potential to shift service use from secondary care towards primary and municipal care | RCT | Yes | Community-dwelling adults aged 65 or older who received home-based-care-services | Whole population | 2464 | Yes |
| González-Franco et al. 2022 | A09 | ES | To analyse the HF-related readmission rate at one month and at 12 months in a group of patients attended to in the Comprehensive Management Units for Patients with HF program compared to another group with similar characteristics that had conventional follow-up. To assess HF patients 12-month mortality rate. | Obs | Yes | People with heart failure | Whole population | 2862 | Yes |
| Karlsson et al. 2013 | A10 | SE | To explore care satisfaction in relation to place of living, HRQoL, functional dependency and health complaints among people 65 years or older, receiving public care and service | C-S | Yes | Older people living at home or in special accommodations | Consecutive | 166 | No |
| Mateo-Abad et al. 2020 | A11 | ES | To evaluate, in the Basque Country, the impact of the CareWell integrated care model for older patients with multimorbidity, using quantitative and qualitative techniques. | Q-E | Yes | Older people with two or more chronic conditions (at least COPD, hearth failure or diabetes mellitus) | Consecutive | 200 | Yes |
| McGloin et al. 2020 | A12 | IE | To describe the impact on hemoglobin A1c (HbA1c), hypoglycemic events, patient empowerment, diabetes distress, and satisfaction with telemonitoring from the patients’ perspective. To explore the perspective of the team regarding the use of using telemonitoring to facilitate the transition to insulin therapy. | M-M | No | Adults with type II diabetes and commencing insulin therapy | Consecutive | 39 | No |
| Ng et al. 2014 | A13 | UK | To determine if the Community Children’s Nursing Outreach Team service, as a model of care, was effective and efficient in its delivery of reducing unscheduled care and admissions to hospital, and improving patient satisfaction | Obs | No | Children | Not specified | 33 | No |
| Phelan et al. 2018 | A14 | IE | To examine the prevalence rates of missed care in the community nursing sector | C-S | No | Public health nurses - community registered general nurses, members of Irish Nurses and Midwives Organization | Purposive | 283 | NA |
| Profili et al. 2017 | A15 | IT | To evaluate the impact of Chronic Care based program on the care of patients with type 2 diabetes | Obs | No | People with type II diabetes | Not specified | 16972 | Yes |
| Röhricht et al. 2017 | A16 | UK | To evaluate the patient satisfaction about the EPC service, three years after the launch | Obs | Yes | People with severe and enduring mental illness | Convenience | 126 | No |
| Senek et al. 2020 | A17 | UK | To contribute to the emerging evidence based on missed care in primary and community care settings | C-S | Yes | Community nurse - district nurse - practice nurse | Not specified | 1742 | NA |
| Smits et al. 2020 | A18 | NL | To provide insight into the safety, efficiency, and patient satisfaction of substituting general practitioners with nurse practitioners for home visits by out-of-hours primary care general practitioner cooperatives. | Q-E | No | People with 24 low complexity health problems* | All patients | 1601 | No |
| Vainieri et al. 2018 | A19 | IT | To investigate how CCM strategies (i.e., education, delivery system and data monitoring system) affect satisfaction and self-management as perceived by patients with single or multiple conditions, both those who are part of a CCM and those who are not. | D | No | People with chronic heart failure or diabetes | Randomized | 1300 | No |
| Vianello et al. 2016 | A20 | IT | To investigate the benefits of a Telemonitoring system in managing Acute Exacerbation in advanced-stage COPD patients to improve their Health-Related Quality of Life and to reduce utilization of healthcare services. | RCT | Yes | People with COPD (Class III-IV) | Consecutive | 334 | Yes |
| Villani et al. 2014 | A21 | IT | To assess the clinical efficacy and cost of this system | RCT | No | People with chronic heart failure | Randomized | 80 | Yes |
| Zimmermann et al. 2016 | A22 | DE | To evaluate the effectiveness of a primary care-based, nurse-led, complex intervention to promote self-management in patients with anxiety, depressive or somatic symptoms. | RCT | Yes | People with anxiety, depressive or somatic symptoms | Consecutive | 325 | No |
| Zúñiga et al. 2015 | A23 | CH | To describe levels and patterns of self-reported implicit rationing of nursing care in Swiss nursing homes. To explore the relationship between staffing level, turnover, and work environment factors and implicit rationing of nursing care. | C-S | Yes | Direct care workers | Randomized | 4307 | NA |

**Legenda. Country:** CH = Switzerland; DE = Germany; Denmark = DK; ES = Spain; FR = France; IE = Ireland; IT = Italy; NL = Netherlands; NO = Norway; SE = Sweden; UK = United Kingdom. **Study Aim(s):** CCM = Chronic Care Model; COPD = Chronic Obstructive Pulmonary Disease; EPC = Enhanced Primary Care; GP = General Practitioner; GSTT = Guy’s and St. Thomas Hospital; HETF **=** Home Enteral Tube Feeding; HF = Heart Failure; HRQoL = Health-Related Quality of Life. **Design:** C-S = Cross-Sectional; D = Descriptive; M-M = Mixed-Methods; Obs = Observational; Q-E = Quasi-Experimental; RCT= Randomized Controlled Trial. **Population:** ACS = Acute Coronary Syndrome. **Comorbidity.** NA = Not applicable.

Table S4. Description of investigated interventions in community care services

| **Study ID** | **Intervention Group** | **Control group** |
| --- | --- | --- |
| **Home Care Services** | | |
| A05 | Two-person team: case manager and primary care physician as single-entry point to refer community-dwelling, very frail patients with complex needs to case managers.  Contents:  *Nursing Interventions*  Until patients were moved to a nursing home or death, nurses as case managers:  - performed home-based comprehensive geriatric assessment  - developed an individualized care plan using evidence-based interdisciplinary protocols  - coordinated all the required services during the follow-up  The role of primary care physician was to collaborate with the case manager in the case management process and to share information on their very frail patients included in the program.  Case manager and primary care physician were supported by geriatricians (in-home specialized needs assessments, implementation of protocols, planned hospital admissions). | Usual care.  Contents:  - medical primary care, in-home visits from primary care physician, nursing, rehabilitation, and social and personal services.  - no integration, no case managers to coordinate all services or community-based geriatricians. |
| A06 | Nurse-led integrated care team.  Contents:  *Multi-professional interventions*  - provide hospital care to patients in their homes or usual place of residence  - provide intensive care with treatments, interventions, and monitoring for a short episode through integrated teamwork with the aim of supporting the patient to return to their previous or an improved health status following an acute episode of ill health. | NA (no control group). |
| A07 | Home Care Centre.  Contents:  *Multi-professional interventions*  - provide clinical services (nursing, specialist and general medicine, rehabilitation, and recovery psycho-physical) and social services. | NA (no control group). |
| A08 | Utilization of the Prevention of Acute Admission Algorithm (PATINA), a prediction model and decisional-support tool to identify patients at risk of hospitalisation.  *Nurse interventions*  - being notified which individuals were at risk of hospitalisation by the PATINA tool every week  - assess the person’s situation using the PATINA decision-support tool within two days | Usual care (not described).  The PATINA algorithm identified patients at risk of hospitalisations but without notifying the Nurse |
| A09 | Follow-up in an intensive and protocolized program.  Contents:  *Multi-professional interventions* (the Comprehensive Management Units for Patients with Heart Failure have nursing staff members)  - provide comprehensive clinical approach with care for heart failure and any comorbidities  - continuing follow-up (in-person visits, telephone contact, and hospital care during the admission)  - formal education on self-care, support measures, commitment of the patient and/or caregiver  - assistance in case of unforeseen decompensation  - direct communication with other specialists.  The follow-up involves both medical and nursing consultations. | Usual care (not described). |
| A10 | Public care and services at home.  Contents  *Nursing Interventions*  -at least two visits at home, by home care nurse (content not described) | Public care and services in special accommodation.  Contents  *Nursing Interventions*  -at least two visits at home, by home care nurse (content not described) |
| A11 | Integrated care pathway based on communication, care coordination, and home-based care.  Contents:  *Multi-professional interventions* (Provided by General Practitioner, the Social Worker, the Specialists, the Nurse Care Manager, and the eHealth Centre)  - identification of frail older patients  - comprehensive baseline assessment  - definition of the therapeutic plan (by phone 24-48h after discharge)  - programmed follow-up (monthly, by phone)  - patient stabilization at home  - integrated care during hospitalization and coordinated hospital discharge  - messaging between patients and/or carers and healthcare practitioners  - patient empowerment program. | Usual care.  Contents:  *Multi-professional interventions* (Provided by Primary Care professionals, GPs and PC nurses)  - well-equipped facilities with 100% coverage of electronic health records (EHR) and e- prescription.  - 24 × 7 eHealth call centre, staffed by trained nurses  - dedicated consultant coordinates other specialists during the hospitalization period. Discharge is coordinated between the hospital liaison nurse and the PC nurse. |
| A13 | Implementation of Community home nursing services, or community children’s nursing outreach teams.  Contents  *Nursing Interventions*  Nursing care (contents not described).  The services, developed to manage acutely ill children at home, were led by the paediatric matron with regular supervision and mentorship of a lead community children’s nursing outreach team’s consultant paediatrician. | Usual care (not described). |
| **Remote Health Services** | | |
| A01 | Teleconsultation performed by stoma nurses.  Contents:  *Nursing Interventions*  Nurses performed the initial clinical examination. Those with specialization in would and stoma care performed follow-up in teleconsultation and arranged appointments. | Usual care.  Contents:  *Nursing Interventions*  Nurses with specialization in would and stoma care or consultants in gastrointestinal surgery performed hospital follow-up consultations. |
| A03 | Nurse-Led Telephone Triage  Contents:  *Nursing Interventions*  - give self-care advice to the patients  - book the patient into a ‘triage-bookable’ face-to-face or telephone appointment with the relevant health professional later the same day or on another day  - book the patient into any routine appointment available  - refer the patient to other NHS services where appropriate, including those outside the practice. | GP-led telephone triage.  Contents:  - give self-care advice to the patients  - book the patient into a ‘triage-bookable’ face-to-face or telephone appointment with the relevant health professional later the same day or on another day  - book the patient into any routine appointment available  - refer the patient to other NHS services where appropriate, including those outside the practice.  Standard Consultation System  -book the patient into an appointment on the same day or on another day  - add patient to the unbooked session |
| A12 | Telemonitoring system in addition to standard care.  Contents:  *Nursing Interventions*  - patients recorded their blood glucose readings as instructed by the CNS  - CNS accessed data for the individual on a prearranged date and contacted the participant, if necessary, to seek additional information on their symptoms and well-being  - patients could contact their CNS or general practitioner if they were worried about their blood glucose readings, as per usual care  - insulin was adjusted according to need using an insulin adjustment plan, as guided by the CNS. | Usual Care.  Contents per patient in the 12-month period following the commencement of insulin therapy:  - from 1 to 3 clinic visits  *Nursing Interventions*  - from 0 to 4 phone calls from the CNS |
| A20 | Telemonitoring system.  Contents:  *Multi-professional interventions*  - Provided to patients a telemonitoring system consisting of a finger pulse-oximeter and a gateway device for data transmission over a telephone line to a central data management unit.  - Explained how to use the kit and provided self-management education materials  - Patients transmitted their monitored heart rate and oxygen saturation values to the operator every other day and/or in the event of subjective clinical worsening  - Out of range values were monitored by a pulmonary specialist who can decide to either modify patient's therapy, send a specialized nurse at home, request an outpatient consultation with a pulmonary specialist or send the patient to the Emergency Department.  *Nursing Interventions*  Nursing home visit within 30 min and 48 h. | Usual care.  Contents:  - if conditions worsen, patients can contact their GP who will manage patient pathway accordingly |
| A21 | Telemonitoring system.  Contents:  Telemonitoring and collection of daily and periodical data from heart failure patients after discharge. The system was designed to communicate with each patient, asking simple questions about their symptoms and giving information and counselling through visual and audible reminders.  *Multi-professional interventions* (provided by cardiologist, psychologist, and nurse)  - The cardiologist decided clinical variables to monitoring and the frequency according to the patients’ characteristics. They also analyzed the information received from each patient.  - The psychologist was available for counseling during each follow-up and evaluated the results of the monthly psychological assessment.  *Nursing Interventions*  -The nurse checked the messages sent by patients and collected patients’ requests. | Usual Care.  Contents:  appointments for follow-up every three months at our Heart Failure clinic after discharge. |
| **Community Health Services** | | |
| A04 | Home Enteral Tube Feeding service in a community clinic. Patients seen at home when possible.  Contents:  *Nursing Interventions*  - meeting patients' nutrition and hydration requirements  - managing common complications  - training and support. | Usual care.  Contents:  *Multi-professional interventions*   - a gastroenterology specialist nurse and dietitian, with a nutrition company nurse providing additional support in the trust. - routine or more urgent support when needed. |
| A14 | Community Health nursing.  Contents:  *Nursing Interventions*   - Nursing care was provided by both Community Registered General Nurses and Public Health Nurses (Contents of nursing care were not described. However, examples of described missed nursing care were health promotion, care management, screening and follow-up, and family support) | NA (no control group). |
| A17 | The study involved nurses who worked in community settings.  *Nursing Interventions*  The description of the content of nursing care were out of the main aim of study. | NA (no control group). |
| A19 | Multidisciplinary teams following the Chronic Care Model composed of GPs and nurses, physiotherapists, dieticians, and medical specialists.  Contents  *Nursing Interventions*  Nurses were responsible for  - contacting patients for routine services  - scheduling specialist visits  - managing individual or group patient counselling  - providing self-management support  - recording patient basic data (such as weight, waist circumference, blood pressure, blood glucose). | NA (no control group). |
| **Primary Health Care** | | |
| A15 | Chronic Care Model.  Contents:  *Multi-professional interventions*   - active role of patients and health education programs - personalized evidence-based therapeutic plan for each patient - scheduled follow-up, shared clinical information, - implementation of primary prevention. - Diabetes patients were periodically monitored for blood pressure, waist circumference, Hb1Ac, cholesterol, electrolytes, urine, blood glucose and microalbuminuria, BMI, lifestyle, eating habits and adherence to the therapy, foot, cardiovascular, ocular, and neurological complications. - Desired parameters were personalized in order to assure monitoring of diabetes and prevent its evolution.   *Nursing Interventions*  The nurse was responsible for data updates, contacting and helping patients for routine services, and carrying out the detection of clinical parameters | Usual practice.  Contents:  Care delivered as provided by LEA |
| A16 | Enhanced primary care service.  Contents:  *Multi-professional interventions* (povided by GP, consultant psychiatrist, senior community psychiatry nurse, social work lead)  - regular GP review  - development of a recovery care plan  - enhanced support to primary care from consultant psychiatrists with regular practice-based mental health multidisciplinary review meetings  - training and education to GPs on managing SMI in primary care  *Nursing Interventions*  - administration of depot medication  - specific assessment of risk factors for physical illness  - signposting into healthy lifestyle services  - training and education to practice nurses on psychopharmacology and therapeutic depot administration | NA (no control group). |
| A18 | Substitution of GP by NP in out-of-hours primary care home visits.  Contents:  *Nursing Interventions*  -Trained NP substituted GP for visiting patients at home during weekday evenings (5:00 PM–11:30 PM) and weekends (10:00 AM–6:00 PM)  - NP took care of 24 low-complex health problems arm complaints, leg complaints, insult, nosebleed, extremity trauma, neck trauma, back trauma, skull trauma, thorax trauma, face trauma, abdominal trauma, burn/inhalation trauma, intoxication, shortness of breath, palpitations, neurological failure, disrupted diabetes, thorax pain, back pain, urinary tract problems, wound, fainting, dizziness, and headache) which could be treated according to a protocol. | GP primary care visits.  Contents:  General Practitioner activities provided following the national guidelines during daytime and out-of-hours primary care |
| A22 | Case management and counselling techniques to promote patients’ self-management.  Contents:  *Multi-professional interventions* (Provided by GP, Nurse, and Psychotherapist)  Primary care-based complex intervention aimed at  - promoting self-management of patients presenting anxiety, depressive or somatic symptoms  - addressing the psychosocial needs of these patients to enhance patients’ self-efficacy  - providing problem-solving techniques  - stimulate relaxation exercises or strengthening self-confidence activities  - case conferences with the GP  - regular meeting among the teams’ members | Usual care (led by GP). Contents:  - medications  - referral for other treatments as well as psychotherapy |
| **Nursing Homes** | | |
| A23 | Nursing homes offer a variety of services, such as long-term care, short stays, adult day care, and post-acute care, including rehabilitation, different home-like environments for people with dementia, gerontopsychiatry, or specialized palliative care.  Contents:  *Nursing Interventions*  The description of the content of nursing care were out of the main aim of study. | NA (no control group). |
| **Transitional Care** | | |
| A02 | Shared care cardiac rehabilitation after acute coronary syndrome at Territorial level.  Contents:  *Multi-professional interventions* (delivered at individual or group level by nurses, physiotherapists, dieticians, and general practitioners), different in their duration and frequency, characterized by:   - Initial and end of the course - Physical exercise - Health education - Smoking cessation - Dietary advice - Risk and clinical evaluation   One visit with cardiologist. | The Hospital-based cardiac rehabilitation was performed entirely within hospital outpatient clinics.  Contents:  *Multi-professional interventions* (delivered at individual or group level by nurses, physiotherapists, dieticians, and cardiologist), different in their duration and frequency, characterized by:   - Initial rehabilitation - Physical exercise - Health education - Smoking cessation Dietary advice - Risk and clinical evaluation at end of rehabilitation   One visit with general practitioner. |

**Legenda.** BMI = Body Mass Index; CNS = Clinical Nurse Specialist; EHR = electronic health records; EPC = Enhanced Primary Care; GP(s) = General Practitioner(s); Hb1Ac = Glycated hemoglobin A; LEA = Essential Level of Assistance; NHS = National Health System; NP = Nurse Practitioner; PC = Primary Care; PC Nurse = Primary Care Nurse; SMI = Severe and enduring mental illness; NA = Not applicable.

**Table S5. Outcomes**

| **Study ID** | **Design** | | **Multidisciplinary** | | **Total (Females)** | | **Age**  **(Mean, ±SD)** | | **IG** | | **CG** | | **Collection tool(s)** | | **Outcome(s)** | | **Key Findings** | |  |
| --- | --- | --- | --- | --- | --- | --- | --- | --- | --- | --- | --- | --- | --- | --- | --- | --- | --- | --- | --- |
| **Home Care Service** | | | | | | | | | | | | | | | | | | |  |
| A05 | Q-E | | Y (with nursing) | | 428  (311) | | -- | | 105 | | 323 | | CD | | Unplanned Hospital Admission | | IG Pre 43 (40,9%) vs. IG Post 8 (14.8%)  CG Post 48 (29.1%)  At 1 year = IG unadjusted OR (95% CI) = 0.46 (0.20-1.06); IG adjusted OR (95% CI) = 0.39 (0.16-0.98) | |  |
| A06 | D | | Y (with nursing) | | 206  (NR) | | -- | | 206 | | NA | | Q | | Patient Satisfaction | | IG Post n = 203 (99.0%) satisfied or very satisfied | |  |
| A07 | C-S | | Y (with nursing) | | 500  (294) | | 81.3 ±10.2 | | 417 | | NA | | Q | | Patient Satisfaction | | IG Post n = 280 (67.2%) trust in the model.  Positive evaluation of the model= 72.1% of city residents and 43% of living in outlying areas (p<0.05).  Near 20.0% of respondents reported organizational deficiencies of the model | |  |
| A08 | RCT | | N (only nursing) | | 2464  (860) | | 85.5 | | 1248 | | 1216 | | CD, IR | | Hospital Readmission occurring 4 to 30 days | | IG hospital readmission n= 20, Incidence per 30 days = 0.016  CG hospital readmission n= 47, Incidence per 30 days = 0.039  Incident Rate Ratio IRR (95%CI) = 0.41 (0.24-0.68), p value = 0.0007 | |  |
| A09 | OBS | | Y (with nursing) | | 2862 (1338) | | -- | | 809 | | 2053 | | NR | | Hospital Readmissions due to Heath Failure | | IG Post at 1 month n = 23 Vs. CG Post at 1 month n = 140  IG Post at 12 months n = 196 Vs CG Post at 12 months n = 647  RR (for readmission IG Vs CG) = 0,436 (95% CI= 0,316-0,600, p<0,001) | |  |
| A10 | C-S | | -- | | 166  (128) | | -- | | 90 | | 76 | | Q | | Patient Satisfaction | | Home (IG) Post= Mean (SD) = 91.7 (9.4) Vs. Special Accommodation (CG) Post Mean (SD) = 85.9 (13.7); p = 0.004. | |  |
| A11 | Q-E | | Y (with nursing) | | 200  (74, 37%) | | 79.4 ±6.8 | | 101 | | 99 | | IR | | Access to Emergency Services | | IG Post Mean (SD) = 0.3 (0.7) Vs. CG Post Mean (SD) = 1.3 (1.6); p ≤0.001 | |  |
|  |  |  |  |  |  |  |  |  |  |  |  |  |  |  | Repeated Hospitalization | | IG Post Mean (SD) = 1.6 (0.7) Vs. CG Post Mean (SD) = 2.3 (1.2) p = 0.008 | |  |
| A13 | OBS | | Y (with nursing) | | 33  (NR) | | -- | | 33 | | -- | | IR | | Access to Emergency Services | | IG Post Vs. CG Post = - 5% | |  |
|  |  |  |  |  |  |  |  |  |  |  |  |  |  |  | Patient Satisfaction | | IG Post 31 patients (94%) described as excellent the care received and 2 (6%) as good | |  |
|  |  |  |  |  |  |  |  |  |  |  |  |  |  |  | Repeated Hospitalization | | CG Post – IG Post = -17.3% | |  |
| **Remote Health Service** | | | | | | | | | | | | | | | | | | |  |
| A01 | RCT | | N (only nursing) | | 110  (55) | | 61.9 ±15.2 | | 52 | | 58 | | Q | | Patient Satisfaction | | IG Good overall experience Yes n = 103 (40.9%) vs No n = 2 (40.0%) vs. CG Good overall experience Yes n = 149 (59.1%) Vs. No n = 3 (60.0%); p 1.000 | |  |
|  |  |  |  |  |  |  |  |  |  |  |  |  |  |  | Self-care | | IG Pre No problems= 5 (14%)  IG Post At 6 months, no problems= 2 (6%) - At 9 months, no problems= 4 (13%) - At >12 months, no problems= 2 (8%)  IG Pre Vs. IG Post % improvement= -6%  CG Pre No problems= 7 (14%)  CG Post At 6 months, no problems= 7 (16%) - At 9 months, no problems= 7 (14%) - At >12 months, no problems= 7 (18%)  CG Pre Vs. CG Post % improvement= 4%  CG Post Vs. IG Post p=0,825 | |  |
| A03 | RCT | | Y (with nursing) | | 16211 | | -- | | 5468 NT | | 5572 GPT  5171 UC | | Q | | Patient Satisfaction | | Patients in the NT arm reported lower overall satisfaction with their care compared with GPT and UC arms. UC – NT = + 3.94 (95% CI 1.88 to 5.99); GPT – NT = + 2.60 (95% CI 0.58 to 4.63) | |  |
| A12 | M-M | | N (only nursing) | | 39  (17, 43%) | | 62.4  (range = 37-80) | | -- | | -- | | Q, I | | Patient Satisfaction | | IG Post all items of Telemedicine Satisfaction and Usefulness Questionnaire were scored above 4 out of 5, indicating a high level of participant satisfaction with the telemonitoring intervention | |  |
|  |  |  |  |  |  |  |  |  |  |  |  |  |  |  | Empowerment | | IG Pre Mean = 3.95 Vs. IG Post Mean = 4.57.  IG Post follow-up Mean = 4.67  IG Pre Vs. IG Post at 3 months, mean difference = +0.72, p<0.001 | |  |
| A20 | RCT | | Y (with nursing) | | 334  (94, 28.1%) | | -- | | 230 | | 104 | | RHD | | Access to Emergency Services | | IG Post Incidence rate per year (95% CI) = 1.29 (1.14-1.46) Vs. CG Post Incidence rate per year (95% CI) = 1.37 (1.14-1.63).  IRR (95% CI) = 0.94 (0.76-1.18), p = 0.58 | |  |
|  |  |  |  |  |  |  |  |  |  |  |  |  |  |  | Repeated Hospitalization | | IG Post Incidence rate per year (95% CI) = 0.11 (0.07-0.16) Vs. CG Post Incidence rate per year (95% CI) = 0.23 (0.14-0.35).  IRR (95% CI) = 0.46 (0.24-0.89), p = 0.01 | |  |
| A21 | RCT | | Y (with nursing) | | 80  (73.7%) | | 72 ±3 | | 40 | | 40 | | Q | | Therapeutic adherence | | IG Pre Morisky scale for compliance to treatment= mean (SD) = 2.8 (0.4) Vs. IG Post (SD) = 3.8 (0.5); p <0.05;  CG Pre Morisky scale for compliance to treatment= mean (SD) = 2.7 (0.9) Vs. CG Post (SD) = 3.0 (0.8); p = ns;  IG Post Morisky scale for compliance to treatment= mean (SD) = 3.8 (0.5) Vs. CG Post (SD) = 3.0 (0.8); p <0.05 | |  |
| **Community Health Services** | | | | | | | | | | | | | | | | | | |  |
| A04 | OBS | | Y (with nursing) | | 100  (NR) | | -- | |  | |  | | IR | | Access to Emergency Services | | Pre-Post Intervention Difference = -93% | |  |
| A14 | C-S | | N (only nursing) | | 283  (277, 98%) | | Range = 25-64 | | 283 | | NA | | Q | | Missed Nursing Care | | Educational attainment was only correlated with ‘report writing’ with those without a degree level qualification more likely to report this activity as missed (p < .05).  Younger community nurses were more likely to miss initial client need assessments (p < .01) and health promotion activities associated with heart disease and stroke, (p < .01).  Community nurses with less than five years’ experience were significantly more likely to miss initial client needs assessments (p < .05), follow-up visits after a re-assessment (p < .01), liaising with other professionals (p < .01), visits with members of the traveller community (p < .05), updating their client notes (p < .05), completing patient notes after a visit (p < .05), other administrative duties (p < .05), visits relating to chronic disease in the community (p < .05), and health promotion work in the community (p < .01) | |  |
| A17 | C-S | | -- | | 1742  (NR) | | -- | | -- | | -- | | Q | | Missed Nursing Care | | Care Left Undone n = 558 (32%)  Community Nurse Understaffed n = 264 (39%) Vs. Full Complement n = 77 (23.5%)  District Nurse Understaffed n = 123 (37.3%) Vs. Full Complement n = 23 (22.1%)  Practice Nurse Understaffed n = 27 (26.5%) Vs. Full Complement n = 46 (21.4%) | |  |
| A19 | D | | Y (with nursing) | | 1300 (52.19% to 57.3%) | | From 72.72 to 74.76 | | -- | | -- | | Q | | Patient Satisfaction | | IG Post Logistic regression models show the not significant relationship between the chronic care model and the patient satisfaction both for single chronic patients (OR = 0.91; p 0.63) and multiple chronic patients (OR = 1.09, p 0.61) | |  |
|  |  |  |  |  |  |  |  |  |  |  |  |  |  |  | Self-care | | Logistic regression models IG and self-care  Single chronic patients (OR = 1.88; p 0.23)  Multiple chronic patients (OR = 0.53, p 0.12) | |  |
| **Primary Health Care** | | | | | | | | | | | | | | | | | | |  |
| A15 | OBS | | Y (with nursing) | | 16972 (8239, 48.5%) | | -- | | 8486 | | 8486 | | RHD | | Access to Emergency Services | | IRR (95% CI) = 0.94 (0.89-1.01), p = 0.085 | |  |
| A16 | OBS | | Y (with nursing) | | 126  (66) | | Mean = 49.2  Range = 26-71 | | -- | | -- | | Q | | Patient Satisfaction | | IG Post  1. Do you believe you are receiving the right treatment/care for you here? Mean = 8.8; SD=1.6 [Range 4-10]  2. Does your named nurse understand you and is she/he engaged in your treatment/care? Mean = 9.0; SD = 1.5 [Range 2-10]  3. Do you believe you are receiving the right medication for you? Mean = 8.9; SD=1.7 [Range 0-10]  4. Do you believe the other elements of treatment/care here are right for you? Mean = 9.4; SD=1.3 [Range 4-10]  5. Do you feel respected and regarded well here? Mean = 9.0; SD=1.5 [Range 4-10] 6. Has treatment/care here been helpful for you? Mean = 9.0; SD=1.4 [Range 4-10] | |  |
| A18 | Q-E | | N (only nursing) | | 513  56.2% | | 74.2 ±15.8  95%CI  73.4–75.0 | | 217 | | 412 | | Q | | Patient Satisfaction | | IG Mean (SD) 8.6/10 (1.0), (range 6–10), 95%CI (8.5-8.8) Vs. CG Mean (SD) 8.3/10 (1.3), (range 1-10), 95% CI (8.2-8.4) | |  |
| A22 | RCT | | Y (with nursing) | | 325  (217) | | -- | | 134 | | 191 | | Q | | Self-efficacy | | IG Pre Mean (SD) = 25.3 (5.6) Vs. IG Post Mean (SD) = 26.8 (6.1)  IG Pre Vs. IG Post Adjusted mean difference (95%CI) = 0.83 (0.15-1.51), p<0.001  IG Post follow-up Mean (SD) = 29.1 (6.1) Vs CG Post follow-up Mean (SD) = 28.1 (6.6)  CG Pre Mean (SD) = 28.6 (5.8) Vs. CG Post Mean (SD) = 28.2 (6.1)  CG Pre Vs. CG Post Adjusted mean difference (95%CI) = 0.25 (-0.31 - 0.82), p >0.05  CG Post Vs. IG Post Adjusted mean difference (95%CI) = -0.58 (-1.48 - 0.33), p >0.05  CG Post follow-up Vs. IG Post follow-up Adjusted mean difference (95%CI) = 1.78 (0.87 - 2.68), p <0.01.  Between group difference from the baseline IG Vs. CG +1.65 points (95% CI 0.52 to 2.78) in favour of IG (p = 0.004) | |  |
| **Nursing Home Service** | | | | | | | | | | | | | | | | | | |  |
| A23 | C-S | | -- | | 4307 (3930, 92.3%) | | 43.37 ±12.21 | | 4307 | | NA | | Q | | Missed Nursing Care | | Care priority is given to support residents in the activities of daily living, such as eating, drinking, elimination, and mobilization.  Nursing activities typically left undone are documentation, social care, rehabilitation, and emotional support.  Full Time Equivalent/100 beds, Staffing and resource adequacy, Teamwork and safety climate, Conflict and lack of recognition, Workload, Lack of preparation were found to be significantly related with implicit rationing of nursing care (p <0.05) | |  |
| **Transitional Care** | | | | | | | | | | | | | | | | | | |  |
| A02 | | RCT | | Y (with nursing) | | 212  (25%) | | -- | | 106 | | 106 | | -- | | Therapeutic adherence | | IG adherence to the Cardiac Rehabilitation program = 53% Vs. CG = 54%  IG Post Vs CG Post RR = 0.98, 95% CI = 0.73–1.32 | |

**Legenda. Design:** C-S = Cross-Sectional; D = Descriptive; M-M = Mixed-Methods; OBS = Observational; Q-E = Quasi-Experimental; RCT= Randomized Controlled Trial. **Multidisciplinary**: Y = Yes; N = No; **Total:** NR = Not Reported; **Sample:** IG = Intervention Group; CG = Control Group; NT = nurse-led computer-supported telephone triage; GPT = GP-led telephone triage; UC = Usual care. **Collection tool(s):** CD = Central Database; I = Interview; IR = Internal Registry; Q = Questionnaire; NR = National Registry; RHD = Regional Health Database. **IG:** IG = Intervention Group; NT = Nurse-led computer-supported Triage;.**CG:** CG = Control Group; GPT = General Practitioner-led telephone Triage; UC = Usual Care; **Key findings:** CG = Control Group; CI = Confidence Interval; IG = Intervention Group; IRR = Incidence Rate Ratio; OR = Odds Ratio; RR = Relative Risk; SD = Standard Deviation. NA = Not applicable

**Table S6. Nursing related determinants of outcomes as reported by authors**

| **Study ID** | **Group** | **Nurse-to-patient-ratio** | **Contract type** | **Skill mix** | **Caseload** | **Staffing model** |
| --- | --- | --- | --- | --- | --- | --- |
| A01 | Intervention | NS | NS | Stoma nurse 1: General practitioner 1: Gastrointestinal surgeon 1 (if needed) | NS | Ten stoma nurses for the whole study |
|  | Control | NS | NS | Stoma nurse 1 or Gastrointestinal surgeon 1 | NS | Ten stoma nurses for the whole study |
| A02 | Intervention | NS | NS | Not clearly stated how many professionals among nurses, dieticians, physiotherapists, rehabilitation nurses, and general practitioners | NS | NS |
|  | Control | NS | NS | Not clearly stated how many professionals among nurses, dieticians, physiotherapists, rehabilitation nurses, and general practitioners | NS | NS |
| A04 | Intervention | NS | NS | Community nutrition nurse | NS | A community nutrition nurse acting multidisciplinary with other services to ensure patient continuing care |
|  | Control | NS | NS | Gastroenterology specialist nurse = 1, Dietician = 1: Nutrition company nurse = 1 | NS | NS |
| A05 | Intervention | 40:1 | NS | Nurse = 1, Primary care physician = 1, Geriatricians n = NS | NS | Not applicable |
| A06 | Intervention | NS | Full-time | NS | NS | 300 patients per month |
| A07 | Intervention | NS | NS | Administrative employee n =1, speech therapist n = 1, physiotherapists n = 3, nurses n = 3, social workers n = 2, doctors in charge of the service n = 2, and medical specialists n = 6 | NS | The Home Care Centre provides services to more than 1600 older people in 26 localities. |
| A12 | Intervention | NS | NS | Only Clinical Nurse Specialist | NS | NS |
| A14 | Intervention | NA | Hours worked per week:  - less than 39, n (%): 83 (29.4%)  - 39, n (%): 121 (42.9)  - more than 39, n (%): 78 (27.7) | NS | Current active clinical caseload:  - 1–50 patients: 125 (45.8%) professionals - 51–100 patients: 68 (24.9%) professionals  - 101–200 patients: 51 (18.7%) professionals - 201–250 patients: 12 (4.4%) professionals - more than 250 patients: 17 (6.2%) professionals  Current active child health caseload (Public Health Nurses only):  - 1–50 patients: 62 (28.8%) professionals - 51–100 patients: 26 (12.1%) professionals - 101–200 patients: 64 (29.8%) professionals - 201–250 patients: 27 (12.6%) professionals  - more than 250 patients: 36 (16.7%) professionals  Current active older person caseload:  - 1–50 patients: 91 (34.0%) professionals - 51–100 patients: 82 (30.6%) professionals - 101–200 patients: 76 (28.4%) professionals - 201–250 patients: 14 (5.2%) professionals - more than 250 patients: 5 (1.9%) professionals | NS |
| A22 | Intervention | 4:125 | Part-time | Nurse 1: General Practitioner 1 | Session provided by ach nurse mean (SD): 133.8 (31.8); range 71-171 sessions | Sessions per patient mean (SD): 4 (2.8); range: 1-17 Duration mean (SD): 53.8 minutes each |
| A23 | Intervention | NS | NS | NS | -Number of full-time equivalents/100 beds, mean (SD): 51.8 (16.0) -Care load (scale from 1 to 12), mean (SD): 5.9 (1.6) | Usual shift: - regular change of shifts, n (%): 1406 (34.4%) - day/evening shift, n (%): 2156 (52.8%) - night shift, n (%): 521 (12.8%) |

**Legenda:** NS = Not specified; SD = Standard Deviation.

**Table S7. Methodological quality according to the study design**

| **Study design** | **STUDY ID** | **Q 1** | **Q 2** | **Q 3** | **Q 4** | **Q 5** | **Q 6** | **Q 7** | | **Q 8** | | **Q 9** | | **Q 10** | | **Q 11** | | **Q 12** | **Q 13** | | **TOTAL** | |
| --- | --- | --- | --- | --- | --- | --- | --- | --- | --- | --- | --- | --- | --- | --- | --- | --- | --- | --- | --- | --- | --- | --- |
| RCT | A01 | YES | NO | YES | NO | NO | NO | YES | | YES | | YES | | YES | | YES | | YES | YES | | **9** | |
|  | A02 | YES | NO | YES | NO | NO | NO | YES | | YES | | YES | | YES | | YES | | YES | YES | | **9** | |
|  | A03 | YES | YES | NO | NO | NO | NO | YES | | YES | | YES | | YES | | YES | | YES | YES | | **9** | |
|  | A08 | YES | NO | YES | UNCLEAR | NO | UNCLEAR | YES | | YES | | YES | | YES | | YES | | YES | YES | | **9** | |
|  | A20 | YES | NO | NO | NO | NO | NO | YES | | YES | | NO | | YES | | NO | | YES | YES | | **7** | |
|  | A21 | NO | YES | YES | NO | YES | NO | YES | | YES | | NO | | YES | | NO | | NO | YES | | **6** | |
|  | A22 | YES | NO | NO | NO | NO | NO | YES | | YES | | YES | | YES | | NO | | YES | YES | | **6** | |
|  | **Notes:** Q1 = Was true randomization used for assignment of participants to treatment groups? Q2 = Was allocation to treatment groups concealed? Q3 = Were treatment groups similar at the baseline? Q4 = Were participants blind to treatment assignment? Q5 = Were those delivering treatment blind to treatment assignment? Q6 = Were outcomes assessors blind to treatment assignment? Q7 = Were treatment groups treated identically other than the intervention of interest? Q8 = Was follow up complete and if not, were differences between groups in terms of their follow up adequately described and analyzed? Q9 = Were participants analyzed in the groups to which they were randomized? Q10 = Were outcomes measured in the same way for treatment groups? Q11 = Were outcomes measured in a reliable way? Q12 = Was appropriate statistical analysis used? Q13 = Was the trial design appropriate, and any deviations from the standard RCT design (individual randomization, parallel groups) accounted for in the conduct and analysis of the trial? Total score is based on the number of questions answered with ‘yes’. | | | | | | | | | | | | | | | | | | | | | |
| Quasi Experimental | **STUDY ID** | **Q 1** | **Q 2** | **Q 3** | **Q 4** | **Q 5** | **Q 6** | **Q 7** | **Q 8** | | **Q 9** | | **TOTAL** | |  | | | | | | |  |
|  | A05 | YES | YES | YES | YES | YES | YES | YES | YES | | YES | | **9** | |  |  |  |  |  |  |  |  |
|  | A11 | YES | YES | YES | YES | YES | YES | YES | YES | | YES | | **9** | |  |  |  |  |  |  |  |  |
|  | A18 | YES | NO | YES | YES | YES | YES | YES | YES | | YES | | **8** | |  |  |  |  |  |  |  |  |
|  | **Notes**: Q1 **=** is it clear in the study what is the ‘cause’ and what is the ‘effect’ (i.e., there is no confusion about which variable comes first)? Q2 = Were the participants included in any comparisons similar? Q3 = Were the participants included in any comparisons receiving similar treatment/care, other than the exposure or intervention of interest? Q4 = Was there a control group? Q5 = Were there multiple measurements of the outcome both pre and post the intervention/exposure? Q6 = Was follow up complete and if not, were differences between groups in terms of their follow up adequately described and analyzed? Q7 = Were the outcomes of participants included in any comparisons measured in the same way? Q8 = Were outcomes measured in a reliable way? Q9 = Was appropriate statistical analysis used? Total score is based on the number of questions answered with ‘yes’. | | | | | | | | | | | | | | | | | | | | | |
| Observational (Cohort studies) | **STUDY ID** | **Q 1** | **Q 2** | **Q 3** | **Q 4** | **Q 5** | **Q 6** | **Q 7** | **Q 8** | | **Q 9** | | **Q 10** | | **Q 11** | | **TOTAL** | | |  | |  |
|  | A12 | YES | YES | YES | NO | NO | NO | YES | YES | | YES | | NO | | YES | | **7** | | |  |  |  |
|  | A04 | NO | YES | UNCLEAR | NO | NO | UNCLEAR | UNCLEAR | YES | | UNCLEAR | | UNCLEAR | | UNCLEAR | | **6** | | |  |  |  |
|  | A09 | YES | YES | YES | YES | NO | YES | YES | YES | | YES | | YES | | YES | | **10** | | |  |  |  |
|  | A16 | NA | NA | YES | NA | NA | YES | YES | YES | | NA | | NA | | YES | | **5** | | |  |  |  |
|  | A15 | YES | YES | YES | YES | YES | YES | YES | YES | | YES | | YES | | YES | | **11** | | |  |  |  |
|  | A13 | UNCLEAR | YES | YES | NO | NO | YES | YES | YES | | NA | | NA | | NO | | **3** | | |  |  |  |
|  | **Notes:** Q1 = Were the two groups similar and recruited from the same population? Q2 = Were the exposures measured similarly to assign people to both exposed and unexposed groups? Q3 = Was the exposure measured in a valid and reliable way? Q4 = Were confounding factors identified? Q5 = Were strategies to deal with confounding factors stated? Q6 = Were the groups/participants free of the outcome at the start of the study (or at the moment of exposure)? Q7 = Were the outcomes measured in a valid and reliable way? Q8 = Was the follow up time reported and sufficient to be long enough for outcomes to occur? Q9 = Was follow up complete, and if not, were the reasons to loss to follow up described and explored? Q10 = Were strategies to address incomplete follow up utilized? Q11 = Was appropriate statistical analysis used? Total score is based on the number of questions answered with ‘yes’. | | | | | | | | | | | | | | | | | | | | | |
| Cross-Sectional | **STUDY ID** | **Q 1** | **Q 2** | **Q 3** | **Q 4** | **Q 5** | **Q 6** | **Q 7** | **Q 8** | | **TOTAL** | |  | | | | | | | | |  |
|  | A07 | NO | YES | YES | YES | YES | YES | YES | YES | | **7** | |  |  |  |  |  |  |  |  |  |  |
|  | A10 | YES | YES | YES | YES | YES | YES | YES | YES | | **8** | |  |  |  |  |  |  |  |  |  |  |
|  | A17 | YES | YES | YES | NO | YES | NO | YES | YES | | **6** | |  |  |  |  |  |  |  |  |  |  |
|  | A23 | YES | YES | YES | YES | YES | NO | YES | YES | | **7** | |  |  |  |  |  |  |  |  |  |  |
|  | A14 | YES | YES | YES | NO | NO | NO | YES | YES | | **5** | |  |  |  |  |  |  |  |  |  |  |
|  | A06 | YES | YES | NO | NO | NO | NO | YES | YES | | **4** | |  |  |  |  |  |  |  |  |  |  |
|  | A19 | YES | YES | YES | YES | YES | YES | YES | YES | | **8** | |  |  |  |  |  |  |  |  |  |  |
|  | **Notes:** Q1 = Were the criteria for inclusion in the sample clearly defined? Q2 = Were the study subjects and the setting described in detail? Q3 = Was the exposure measured in a valid and reliable way? Q4 = Were objective, standard criteria used for measurement of the condition? Q5 = Were confounding factors identified? Q6 = Were strategies to deal with confounding factors stated? Q7 = Were the outcomes measured in a valid and reliable way? Q8 = Was appropriate statistical analysis used? Total score is based on the number of questions answered with ‘yes’. | | | | | | | | | | | | | | | | | | | | | |
